# Calcium–phosphate metabolism in patients with multiple sclerosis

**DOI:** 10.1007/s40618-014-0235-x

**Published:** 2015-01-18

**Authors:** K. Kubicka-Baczyk, B. Labuz-Roszak, K. Pierzchala, M. Adamczyk-Sowa, A. Machowska-Majchrzak

**Affiliations:** 1 Clinical Department and Chair of Neurology in Zabrze, Medical University of Silesia, Katowice, Poland; 2 Clinical Department and Chair of Neurology in Zabrze, Medical University of Silesia, 3-go Maja 13/15, 41-800 Zabrze, Poland

**Keywords:** Relapsing–remitting sclerosis multiplex, Calcium–phosphate metabolism, 25-Hydroxycholecalciferol

## Abstract

**Introduction and objectives:**

The purpose of this study was to evaluate the concentration of 25-hydroxycholecalciferol and parameters of calcium–phosphate metabolism at different periods of relapsing–remitting multiple sclerosis (RRMS).

**Materials and methods:**

Forty-five patients, residents of Poland (49°–50°, N), were enrolled in the study, i.e. 15 immediately after the diagnosis of RRMS, 15 at the early stage and 15 at the advanced stage of RRMS. The results were compared to values obtained in 20 age- and sex-matched controls.

**Results:**

Lower serum concentrations of 25-hydroxycholecalciferol and ionised calcium were found in patients compared to the control group. In patients with the disease duration of 5–6 years, concentrations of 25-hydroxycholecalciferol and ionised calcium were lower than in patients in the earlier period of RRMS. The inverse and clearer direction of changes was found in parathormone serum concentration in patients compared to the controls. In patients with a longer disease duration, a significantly lower 25-hydroxycholecalciferol concentration was found in female patients compared to male patients. In patients, more frequent 25-hydroxycholecalciferol and unsaturated fatty acids’ supplementation was observed compared to the controls.

**Conclusions:**

In RRMS patients, calcium–phosphate metabolism is disturbed which increases during disease progression.

## Introduction

Multiple sclerosis (MS) is a chronic inflammatory-demyelinating disease of the central nervous system (CNS). The aetiology of the disease is not entirely understood. It is characterised by multifocal and disseminated in time damage to the CNS with heterogeneous symptomatology and the clinical course. Depending on the studied region, the prevalence in the general population varies from 5 to 150 per 100,000 individuals [1].

The first symptoms usually appear between the ages of 20 and 40 [2, 3] and the disease is the most frequently observed among Caucasians, with a higher prevalence in females [4].

Currently, MS is a disease of complex pathogenesis with genetic, immunopathological and environmental factors [4, 5]. Epidemiologic studies suggest the prevalence rate increase at higher latitudes [6]. The protective effect of sunlight seems to be related to the protective action of 25(OH)D_3_, which plays an important role in immunological processes and in the regulation of calcium–phosphate metabolism.

Recent studies have demonstrated that 25(OH)D_3_ participates in cell proliferation and differentiation, and also in immunological phenomena [7].

The greatest 25(OH)D_3_ deficiency was noted in the spring and in the winter and also in the elderly. Females aged 15–45 years are at a greater risk of 25(OH)D_3_ deficiency, especially in combination with seasonal variations. The peak of incidence is noted between the third and the fourth decades of life [8, 9].

To provide complex medical care, it is important to determine the indices of calcium–phosphate metabolism in patients with MS.

### The purpose of the study

The study evaluated the concentration of 25(OH)D_3_ and the indices of calcium–phosphate metabolism depending on the sex, number of relapses, degree of motor disability and the applied treatment at different stages of relapsing–remitting MS (RRMS).

## Materials and methods

Patients with diagnosed RRMS were enrolled in the study, according to the McDonald criteria of 2005. The patients were selected based on the medical documentation of the out-patient neurological clinic and the medical history of patients hospitalised between 2009 and 2011 in the Clinic of Neurology in Zabrze, Poland.

The enrolment criteria were as follows: diagnosis of RRMS according to the McDonald criteria; the current degree of disability according to the Expanded Disability Status Scale (EDSS) from 0.0 to 3.0 points; inhabitation of the Silesian agglomeration (at a latitude of 49°–50°, N); performance of the professional work under similar environmental conditions; absence of any other chronic disease except MS, patients’ informed written consent for the study. Recruited patients had not followed any diet which might have influenced the calcium–phosphate metabolism (e.g. diabetic diet, weight-reduction diet, high-protein diet, etc.). Patients had not consumed dietary supplements with vitamin D before or at the time of the study, and within 6 months prior to sample collection had not resided in other climatic zones.

The exclusion criteria were as follows: CNS demyelinating syndrome of different aetiology than MS; forms of MS other than RRMS; duration of disease longer than 6 years and the degree of motor disability >3.0 points according to the EDSS; relapse at the time of examination; treatment with immunomodulatory agents or glucocorticoids (GCs) for the mean duration of 6 or 12 weeks depending on the qualification to a study subgroup; diagnosed kidney disease, gastrointestinal tract and liver diseases, endocrine disorders, menopausal women.

The results were compared to values obtained in 20 age- and sex-matched controls who did not differ in terms of place of residence or ethnicity.

The study was approved by the Bioethics Committee of the Medical University of Silesia in Katowice, Poland.

All enrolled patients were interviewed with particular attention paid to the course of RRMS: onset of symptoms, year of diagnosis, additional tests, number of relapses (exacerbations of the disease manifested with new neurological symptoms), hospitalisations, previous treatment, family and occupational history and comorbidities. General and neurological examination was conducted and the degree of motor disability of patients was evaluated using the EDSS [10].

In every patient, 5 ml fasting blood sample was obtained and daily (24 h) urine sample was collected. Laboratory tests evaluated the indices of calcium–phosphate metabolism, the results of which were compared to the applicable standards for specific test methods.

Serum concentration of 25(OH)D_3_ (normal range 11.1–42.9 ng/ml; 30.4–118.5 nmol/l) and parathormone concentration (normal range 15–65 pg/ml) were assessed using the COBAS e 601 analyser (Roche).

Serum phosphorus concentration (normal range 0.81–1.45 mmol/l), serum calcium concentration (normal range 2.1–2.55 mmol/l), alkaline phosphatase concentration (normal range 30–90 U/l), bone alkaline phosphatase concentration (normal range 20–48 U/l), and concentration of daily urine calcium excretion (normal range 2.5–6.5 mmol/l/24 h) were assessed using the COBAS c 501 analyser (Roche).

Ionised calcium concentration (normal range: 1.13–1.32 mmol/l) was measured directly using ion-selective electrodes (RAPID lab 865, Siemens).

Samples for laboratory analyses were collected in April and the sun exposure was similar for all the examined subjects.

The results were stored in the database prepared specifically for this purpose in Microsoft EXCEL. STATISTICA 9.1 was used for the statistical analysis. The result of the statistical analysis was considered statistically significant if the level of significance was *p* ≤ 0.05. In data description, the standard statistical parameters were provided, i.e. the number *N*, arithmetic mean *X*, the SD, median and percentages (%). Normal distribution of data was assessed by the Shapiro–Wilk normality test. Statistical significance of between-group differences was verified by the Mann–Whitney *U* test and the Kruskal–Wallis test. The Chi-squared test was utilised for comparison of qualified variables.

## Results

Forty-five patients with RRMS were divided into three groups. The results were compared with the control group (Table 1).

**Table 1:** The characteristics of the study groups

	Group 1	Group 2	Group 3	Control group
Number of subjects (*N*)	15	15	15	20
Stage of disease	Immediately after the diagnosis of RRMS	In the early stage of RRMS up to 6 months after the occurrence of first symptoms	In the advanced stage of RRMS 5–6 years after the occurrence of first symptoms	Healthy individuals, not diagnosed with any CNS disease or any other systemic or metabolic disorders
Treatment used	Not treated with immuno-modulatory agents and GCs	Subjects not used immunomodulatory agents and were not treated with GCs 6 weeks prior to study enrolment	Subjects not used immuno-modulatory agents and were not treated with GCs 12 weeks prior to study enrolment	Not treated with immuno-modulatory agents or GCs
Sex (F/M)*	9 (60 %)/6 (40 %)	10 (66.7 %)/5 (33.3 %)	12 (80 %)/3 (20 %)	14 (70 %)/6 (30 %)
Age (years)**	35.3 ± 12.1	33.5 ± 8.9	36.2 ± 6.1	35.2 ± 8.2
EDSS (points)**	1.2 ± 0.65	1.2 ± 0.44	1.8 ± 0.7	–
Number of relapses before the enrolment to the study**	1.3 ± 1.1	2.4 ± 2.1	3.5 ± 2.4	–

*RRMS* relapsing–remitting multiple sclerosis, *GCs* glucocorticoids, *CNS* central nervous system, *F* female, M male *EDSS* Expended Disability Status Scale

* Data presented as *n* (%)

** Data presented as mean ± SD

In the studied groups, serum ionised calcium concentrations differed significantly (*p* = 0.002). The most significant differences regarding the concentrations of ionised calcium were observed between group 1 and group 2 (*p* = 0.004), and between group 1 and the individuals in the control group who had higher concentrations compared to patients in group 1 (*p* = 0.003) (Tables 2, 3).

**Table 2:** Laboratory results in the examined groups

Parameter	Statistical parameter	Group 1 *N* = 15	Group 2 *N* = 15	Group 3 *N* = 15	Control group *N* = 20	Kruskal–Wallis test
Serum phosphorus (mmol/l)	*X*	1.07	1.13	1.01	1.06	NS (*p* = 0.130)
	SD	0.23	0.2	0.17	0.16	
	Me	1.05	1.2	1.03	1.07	
	10 % P	0.87	0.93	0.88	0.9	
	90 % P	1.19	1.31	1.18	1.23	
Serum calcium (mmol/l)	*X*	2.38	2.37	2.37	2.32	NS (*p* = 0.160)
	SD	0.14	0.2	0.08	0.07	
	Me	2.40	2.36	2.39	2.31	
	10 % P	2.20	2.25	2.25	2.25	
	90 % P	2.56	2.44	2.45	2.42	
Serum alkaline phosphatase (U/l)	*X*	57.2	63.9	62	56.7	NS (*p* = 0.240)
	SD	10.7	16.8	18.7	22.1	
	Me	55	59	56	49	
	10 % P	45.4	43.4	44.8	38.9	
	90 % P	72.8	85	88.2	85.4	
Serum bone alkaline phosphatase (U/l)	*X*	21.5	22.8	22.1	21.8	NS (*p* = 0.900)
	SD	8.1	7.7	7.2	9.6	
	Me	22	23	21	20.5	
	10 % P	12	12.4	15.4	11	
	90 % P	26.6	32.2	31.2	32.2	
Serum ionised calcium (mmol/l)	*X*	1.1	1.14	1.09	1.18	***p*** **=** **0.002**
	SD	0.21	0.06	0.05	0.17	
	Me	1.07	1.14	1.08	1.14	
	10 % P	0.99	1.07	1.05	1.06	
	90 % P	1.14	1.2	1.14	1.28	
Serum parathormone (pg/ml)	*X*	24.33	61.01	76.7	52.28	***p*** **=** **0.00001**
	SD	11.25	34.97	41.97	23.68	
	Me	22.59	60.1	64.39	48.93	
	10 % P	7.36	16.06	37.11	27.24	
	90 % P	36.86	103.22	11582	82.88	
Serum 25(OH)D_3_ (ng/ml)	*X*	20.3	16.91	13.89	26.28	***p*** **=** **0.007**
	SD	10.38	8.44	6.48	8.24	
	Me	17.75	15.59	13.4	28.09	
	10 % P	9.5	7.41	5.76	16.69	
	90 % P	31.81	29.29	21.11	38.33	
Daily urine calcium excretion (mmol/l/24 h)	*X*	2.36	2.67	3.13	3.79	NS (*p* = 0.310)
	SD	0.98	1.9	2.53	2.68	
	Me	2.16	1.86	1.67	3.04	
	10 % P	1.21	1.26	1.29	1.17	
	90 % P	3.57	5.13	7.47	7.42	

*X* mean, *SD* standard deviation, *Me* median, *10* *% P, 90* *% P* percentiles, *NS* not significant, *P* level of significance

**Table 3:** The comparisons between pairs of groups for serum ionised calcium, parathormone and 25(OH)D_3_ concentration values (Mann–Whitney *U* test)

Comparison	Ionised calcium	Parathormone	25(OH)D_3_
Group 1–group 2	***p*** **=** **0.004**	***p*** **<** **0.001**	NS (*p* = 0.300)
Group 1–group 3	NS (*p* = 0.250)	***p*** **<** **0.001**	NS (*p* = 0.090)
Group 1–control group	***p*** **=** **0.003**	***p*** **<** **0.001**	NS (*p* = 0.060)
Group 2–group 3	***p*** **=** **0.020**	NS (*p* = 0.590)	NS (*p* = 0.490)
Group 2–control group	NS (*p* = 0.880)	NS (*p* = 0.230)	***p*** **=** **0.002**
Group 3–control group	***p*** **=** **0.020**	***p*** **=** **0.050**	***p*** **<** **0.001**

Significant values (*p* ≤ 0.05) are in bold

An association was found between the mean parathormone concentrations and the disease duration. In group 3, the mean serum concentration of parathormone was the highest and was above the upper limit of the reference range. Newly diagnosed patients had the lowest serum concentration of parathormone compared to serum concentration of parathormone in patients at a more advanced stage of the disease as well as in the control group (Table 2).

A decrease in mean concentrations of 25(OH)D_3_ with the disease duration was noted. The mean serum concentration of 25(OH)D_3_ in the studied groups of patients was lower compared to the control group (*p* = 0.007). A significant difference in concentrations was demonstrated between the control group and patients with the disease duration of up to 6 months (*p* = 0.002) or longer (*p* **<** 0.001) (Tables 2, 3).

Next, the indices of calcium–phosphate metabolism were assessed depending on the sex. In group 3 (patients with longer disease duration), lower concentrations of 25(OH)D_3_ were found in females compared to males. In the group of patients with a shorter disease duration (groups 1 and 2), the mean serum concentration of 25(OH)D_3_ was higher in females compared to males with a similar degree of motor disability. The mean serum concentrations of parathormone in male patients at the advanced stage of the disease (group 3) were higher compared to females. Both in females in group 2 and in all patients in group 3, regardless of the sex, the mean concentrations of parathormone exceeded the upper limit of the reference range (Table 4).

**Table 4:** Parameters of calcium–phosphate metabolism in the examined groups, depending on sex (mean ± SD)

Parameter	Sex	Group 1 (*N* = 15)	Group 2 (*N* = 15)	Group 3 (*N* = 15)	Control group (*N* = 20)
Serum phosphorus (mmol/l)	M	1.10 ± 0.35	1.07 ± 0.29	1.03 ± 0.17	0.94 ± 0.14
	F	1.06 ± 0.12	1.17 ± 0.15	1.00 ± 0.17	1.11 ± 0.14
Serum calcium (mmol/l)	M	2.41 ± 0.10	2.35 ± 0.05	2.39 ± 0.04	2.35 ± 0.10
	F	2.37 ± 0.16	2.37 ± 0.25	2.37 ± 0.08	2.31 ± 0.06
Serum alkaline phosphatase (U/l)	M	63.2 ± 12.7	66.0 ± 21.5	66.0 ± 28.0	76.2 ± 24.3
	F	53.2 ± 7.9	62.8 ± 15.2	61.0 ± 17.3	48.4 ± 15.3
Serum bone alkaline phosphatase (U/l)	M	20.7 ± 8.0	21.6 ± 6.9	26.3 ± 3.2	28.5 ± 12.1
	F	22.1 ± 8.7	23.4 ± 8.4	21.0 ± 7.6	18.9 ± 7.0
Serum ionised calcium (mmol/l)	M	1.03 ± 0.04	1.13 ± 0.04	1.06 ± 0.01	1.15 ± 0.12
	F	1.14 ± 0.27	1.15 ± 0.08	1.09 ± 0.06	1.19 ± 0.19
Serum parathormone (pg/ml)	M	22.72 ± 9.34	47.03 ± 40.56	83.18 ± 48.22	51.55 ± 19.61
	F	25.40 ± 12.80	68.00 ± 31.76	75.07 ± 42.48	52.59 ± 25.92
Serum 25(OH)D_3_ (ng/ml)	M	18.22 ± 7.04	14.61 ± 10.35	17.45 ± 6.11	24.48 ± 9.38
	F	21.70 ± 12.34	18.05 ± 7.67	13.00 ± 6.50	27.05 ± 7.95
Daily urine calcium excretion (mmol/l/24 h)	M	2.24 ± 0.99	4.16 ± 2.63	2.66 ± 1.34	4.76 ± 1.88
	F	2.44 ± 1.02	1.92 ± 0.84	3.25 ± 2.78	3.37 ± 2.92

*M* male, *F* female

The increase in serum parathormone concentration was observed with the increase in relapses (*R* = 0.51, *p* < 0.001) in the conducted correlation analysis between the number of relapses, the degree of disability and the indices of calcium–phosphate metabolism in all patients. No other significant correlation was found.

## Discussion

The present study showed that in patients with RRMS, serum concentrations of 25(OH)D_3_ and ionised calcium were significantly lower compared to healthy individuals and decreased with the duration of the disease, with the increase in relapses and in females compared to males. However, the concentration of parathormone increased significantly as RRMS progressed.

Our results concerning the concentration of 25(OH)D_3_ in RRMS patients are compatible with those of some [10, 11], but not all [12–15] prior publications (Table 5). Moen et al. [12], Kragt et al. [13], Soilu-Hanninen et al. [14], and Barnes et al. [15] presented different results obtained in Norway, the Netherlands, Finland, Great Britain and Northern Ireland. The researchers showed comparable mean 25(OH)D_3_ concentrations in patients with MS and healthy persons.

**Table 5:** Summary table with data from the past publications and the present study

	Kubicka-Baczyk et al.	Past publications							
		Smolders et al.	Van der Mei et al.	Hussein et al.	Moen et al.	Pierrot-Deseilligny et al.	Kragt et al.	Barnes et al.	El-Ghoneimy et al.
Place of the study	Poland region Silesia	Maastricht, Netherlands	Tasmania, Australia	Cair, Egypt	Oslo, Norway	Paris, France	Amsterdam, Netherlands	Ulster, Northern Ireland	Cair, Egypt
Latitude	49°–50°N	50°–51°N	41°–43°S	30°N	59°55′N	48°52′N	52°22′N	54°35′N	30°N
Number of subjects (N)	65	43	89	35	99	76	101	29	20
Age (years)*	35.0 ± 9.0	34.1 ± 10.1	43.5 ± 9.3	–	–	41 ± 10	45.0 ± 11.9	46.79 ± 11.3	28.7 ± 6.0
EDSS (points)	1.41 ± 0.69	2.68 ± 1.74	3.5 ± 2.2	4.09 ± 1.9	1.4 ± 1.1	2.3	4	–	3.4 ± 2.3
Serum 25(OH)D_3_ (nmol/l)*	54.16 ± 26.81 ↓	79.47 ± 32.2 ND	51.4 ± 20.3 ≈	33.65 ± 22.7 ↓	68.4 ± 24.5 ≈	49 ± 22 ND	78.65 ± 29.6 ≈	69.19 ± 40.0 ≈	31.8 ± 14.31 ND
Serum parathormone (pmol/l)*	82.64 ± 44.98 ↑	–	–	–	3.55 ± 1.89 ≈	–	5.35 ± 1.85 ≈	61.18 ± 26.01 ≈	65.24 ± 37.3 ND
Serum ionised calcium (mmol/l)*	1.11 ± 0.11 ↓	–	–	–	1.27 ± 0.03 ≈	–	2.36 ± 0.09 ≈	–	0.66 ± 0.29 ND

* Data presented as mean ± SD, “↓” results in MS patients were significantly lower than in controls, “↑” results in MS patients were significantly higher than in controls. “≈” results in MS patients were comparable to controls; *ND* not defined (results in MS patients were not compared to controls), “–” not studied

In the present study, the relationship between 25(OH)D_3_ concentration and the degree of motor disability according to the EDSS was also analysed and no correlation was found. Contrary results were obtained by Van der Mei et al. [10], Hussein et al. [16] and Smolders et al. [17]. Patients with a higher serum concentration of 25(OH)D_3_ had a better motor ability.

In the present study, an increase was observed in the mean concentration of parathormone with disease duration and the simultaneous decrease in the mean concentration of 25(OH)D_3_.

However, the study results of the Soilu-Hanninen et al. were different. They showed lower serum concentrations of parathormone and ionised calcium in patients with RRMS compared to healthy persons. In the present study, the concentrations of mineral metabolism indices (i.e. phosphorus, alkaline phosphatase and bone alkaline phosphatase) were within the reference range, which is consistent with the results obtained in Scandinavia and France [15, 18].

In the present study in patients with RRMS diagnosed within 6 months and about 6 years prior to the study, higher concentrations of parathormone and ionised calcium were found and lower 25(OH)D_3_ concentrations were observed compared to patients with the shortest disease duration. This would indicate the influence of disease duration on the examined indices. A group of patients with RRMS in whom an increased parathormone concentration was observed was in remission. This is contrary to the results of Soilu-Hanninen et al. [7] and Pierrot-Deseilligny et al. [14] who examined the mineral metabolism in patients during an MS relapse.

Due to the fact that MS occurs more frequently in females, in the present study the attention was paid to the concentration of the examined indices, depending on the sex of patients. According to Kuchuk et al. [19], the largest deficiency of 25(OH)D_3_ in females appears during the greatest fertility between the ages of 15 and 45 years. The peak of prevalence for MS occurs at the same time.

Analysing the data from the present study, a significantly lower serum concentration of 25(OH)D_3_ was observed in the course of disease progression. Moreover, in the group of patients with a longer disease duration a significantly lower 25(OH)D_3_ concentration in females was detected compared to male patients with the similar degree of motor disability.

Hussein et al. [13], Kragt et al. [16] and El-Ghoneimy et al. [20] evaluated 25(OH)D_3_ concentration depending on the sex of individuals inhabiting regions of different sun exposure. Contrary to the results of the present study, Hussein et al. [16] and El-Ghoneimy et al. [20] demonstrated a higher concentration of 25(OH)D_3_ in healthy males compared to females. The authors are of the opinion that the obtained results may be determined by cultural and religious factors of the communities inhabiting Arabic countries.

## Conclusions

In patients with RRMS, serum concentrations of 25(OH)D_3_ and ionised calcium were significantly lower compared to healthy individuals and decreased with the duration of the disease, with the increase in relapses and in females compared to males. However, the concentration of parathormone increased significantly as RRSM progressed.

Due to the geographical location, Poland is one of the countries with a high risk of MS incidence. However, studies on calcium–phosphate metabolism have not been conducted in Poland as yet.

The role of the deficiency of 25(OH)D_3_ in patients with MS immediately after the diagnosis and/or the increasing 25(OH)D_3_ deficiency in the course of disease progression requires further research as well as the determination of indices of mineral metabolism depending on the treatment used and in the relapse or remission.

The results of the present study and the available literature show that it is appropriate to determine the indices of calcium–phosphate metabolism, including 25(OH)D_3_ in patients immediately after diagnosing MS and in the course of disease progression. Depending on the results, 25(OH)D_3_ supplementation should be considered.

### Limitation of the study

The limitation of the present study is a small number of patients with RRMS and healthy controls. 25(OH)D2 was not measured. Further study is planned.

## Abbreviations

25(OH)D_3_: 25-Hydroxycholecalciferol
MS: Multiple sclerosis
RRMS: Relapsing–remitting multiple sclerosis
CNS: Central nervous system
IL: Interleukin
UVB: Ultraviolet-B radiation
GCs: Glucocorticoids
EDSS: Expanded Disability Status Scale
SD: Standard deviation
